# Gut microbiota in heart failure and related interventions

**DOI:** 10.1002/imt2.125

**Published:** 2023-07-10

**Authors:** An‐Tian Chen, Jian Zhang, Yuhui Zhang

**Affiliations:** ^1^ Department of Cardiology, State Key Laboratory of Cardiovascular Disease, Fuwai Hospital Chinese Academy of Medical Sciences & Peking Union Medical College/National Center for Cardiovascular Diseases Beijing China; ^2^ State Key Laboratory of Cardiovascular Disease, Heart Failure Center, Fuwai Hospital Chinese Academy of Medical Sciences & Peking Union Medical College/National Center for Cardiovascular Diseases Beijing China; ^3^ Key Laboratory of Clinical Research for Cardiovascular Medications National Health Committee Beijing China

**Keywords:** gut microbiota, heart failure, short‐chain fatty acids, trimethylamine N‐oxide

## Abstract

Heart failure (HF) is a sophisticated syndrome with structural or functional impairment of ventricular filling or ejection of blood, either causing symptoms and signs or being asymptomatic. HF is a major global health issue affecting about 64.3 million people worldwide. The gut microbiota refers to the complex ecosystem of microorganisms, mainly bacteria, in the gut. Studies have revealed that the gut microbiota is associated with many diseases ranging from neurodegenerative diseases to inflammatory bowel disease and cardiovascular diseases. The gut hypothesis of HF suggests that low cardiac output and systemic circulation congestion would cause insufficient intestinal perfusion, leading to ischemia and intestinal barrier dysfunction. The resulting bacterial translocation would contribute to inflammation. Recent studies have refined the hypothesis that changes of metabolites in the gut microbiota have a close relationship with HF. Thus, the gut microbiota has emerged as a potential therapeutic target for HF due to both its critical role in regulating host physiology and metabolism and its pivotal role in the development of HF. This review article aims to provide an overview of the current understanding of the gut microbiota's involvement in HF, including the introduction of the gut hypothesis of HF, its association with HF progression, the potential mechanisms involved mediated by the gut microbiota metabolites, and the impact of various interventions on the gut microbiota, including dietary interventions, probiotic therapy, fecal microbiota transplantation, antibiotics, and so on. While the gut hypothesis of HF is refined with up‐to‐date knowledge and the gut microbiota presents a promising target for HF therapy, further research is still needed to further understand the underlying mechanisms between gut microbiota and HF, the efficacy of these interventions, and contribute to the health of HF patients.

## Highlights

- Heart failure is associated with the gut microbiota, while the gut hypothesis of heart failure is less discussed in a thorough manner.
- We reviewed how changes in metabolites of the gut microbiota contribute to heart failure and possible underlying mechanisms.
- We also reviewed potential interventions for heart failure targeting the gut microbiota, including dietary interventions, probiotic therapy, fecal microbiota transplantation, antibiotics, and other approaches.

## Graphical abstract

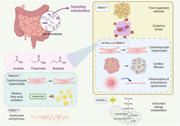

## INTRODUCTION

Research on the gut microbiota has been flourishing in recent years, with numerous studies reporting its relationship with diseases such as type 2 diabetes, obesity, fatty liver disease, gastrointestinal diseases, and certain types of cancer [1]. Worldwide, there are approximately 64.3 million heart failure (HF) patients, with HF patients accounting for 1%–2% of adults in developed countries [2, 3]. The definition of HF was reached in 1983, it is suggested that “Heart failure is the state of any heart disease in which, despite adequate ventricular filling, the heart's output is decreased or in which the heart is unable to pump blood at a rate adequate for satisfying the requirements of the tissues with function parameters remaining within normal limits” [4]. The 2022 American Heart Association/American College of Cardiology/Heart Failure Society of America (AHA/ACC/HFSA) guideline indicates that “HF is a complex clinical syndrome with symptoms and signs that result from any structural or functional impairment of ventricular filling or ejection of blood,” and asymptomatic stages with either cardiomyopathies or structural heart disease are considered at‐risk for HF or pre‐HF [5]. HF is characterized by circulatory congestion, which can lead to intestinal swelling and damage to the intestinal barrier. This can exacerbate inflammation through bacterial translocation, highlighting the potential role of the gut microbiota in HF [6].

Prevention of HF is important, especially for patients at at‐risk for HF or pre‐HF. Primary preventions cover controlling blood pressure, usage of sodium‐glucose cotransporter 2 inhibitors (SGLT2i) for patients with type 2 diabetes, and a healthy lifestyle with no cigarettes [5]. Treatments for HF mainly include pharmacological treatment, device and interventional therapies, mechanical circulatory support (MCS), and heart transplantation [5]. Commonly used drugs contain renin–angiotensin system inhibitors such as angiotensin‐converting enzyme inhibitors (ACEi) or angiotensin (II) receptor blockers (ARB) or angiotensin receptor‐neprilysin inhibitors (ARNi), beta‐blockers, mineralocorticoid receptor antagonists, SGLT2i, hydralazine, isosorbide dinitrate, and other drugs [5]. Device and interventional therapies mainly refer to implantable cardioverter defibrillator and cardiac resynchronization therapy to prevent sudden cardiac death. The most widely used MCS is the left ventricular assist device, which is regarded as both a bridge to transplantation and as destination therapy [7]. End‐stage HF patients satisfying certain criteria may undergo heart transplantation [8].

According to an estimation, the microbes in bodies could collectively consist of as many as 100 trillion cells, which is 10‐fold the number of human cells. It is also pointed out that microbes could encode 100‐fold more unique genes than the human genome [9]. It is believed that the gut holds the majority of microbes [10]. By examining fecal samples of 124 European individuals, it is found that each habored at least 160 bacterial species [11]. Besides HF, the gut microbiota is closely related to many diseases, including autism spectrum disorder [12], neurodegenerative diseases such as Alzheimer's disease [13] and Parkinson's disease [14], inflammatory bowel disease (IBD) [15], cardiovascular diseases (CVDs) such as atherosclerosis [16] and ischemic heart disease [17], and so on. As for HF, the gut microbiota acts like an endocrine organ, as several metabolites generated by its metabolism are involved in the disease status of HF [6].

The whole story originated from the gut hypothesis. By searching the Pubmed with keywords “(gut hypothesis) AND (heart failure),” the first article discussing the gut hypothesis is in 1999 by Niebauer et al. [18]. Actually, the concept of the gut hypothesis in heart failure could be traced back to no later than 1997, implying the role of chronic heart failure (CHF) in leading to increased bowel permeability and consequently bacterial translocation and release of endotoxin [19]. Overall, the inflammatory response would be triggered in HF patients [20]. After that, more researchers focused on the relationship between the gut microbiota and HF. To explore the complex interactions between the gut microbiota, a series of studies have been performed in discovering changes in the gut microbiota in HF patients [21], researching the effects of metabolites by the gut microbiota in contributing to HF [22], and possible interventions concerning the gut microbiota or the metabolites in alleviating HF [23]. Metabolites, mainly trimethylamine N‐oxide (TMAO) and short‐chain fatty acids (SCFAs), play an important role in the interaction with HF. Certain daily diet intakes will be transformed into trimethylamine (TMA) by the gut microbiota and eventually converted into TMAO in the liver. SCFAs are generated from dietary fibers and contribute to providing energy to the failing heart [24]. Other substances including N,N,Ntrimethyl‐5‐aminovaleric acid (TMAVA) is also produced during gut metabolism from trimethyllysine and contribute to HF.

In this review, we aim to briefly introduce “the gut hypothesis of heart failure,” the role of gut microbiota metabolites in HF, and related interventions.

## GUT HYPOTHESIS OF HEART FAILURE AND IMPAIRMENT OF INTESTINAL BARRIER

The “gut hypothesis of HF” has gained popularity in recent years, with increasing amounts of studies supporting its validity. This hypothesis proposes that low cardiac output and circulation congestion in the system lead to reduced intestinal perfusion, resulting in ischemia and damage to the intestinal barrier. The intestinal barrier is mainly formed by the intestinal epithelium mechanically linked with each adjacent cell with selective permeability to enable the absorption of nutrients, electrolytes, and water, and disable the invasion of toxins, antigens, and enteric flora [25]. Ischemia of the intestines would generate a series of pathological changes such as transmural necrosis and reversible mucosal injury, and the severeness depends on the severity and duration of ischemia, whether it is occlusive or non‐occlusive, where and to what content is occluded and how long it takes to start pathological examination after ischemia [26]. In severe cases, digestive enzymes may enter the intestinal wall during the ischemic period as a result of intestinal barrier dysfunction [27].

**Figure 1:**
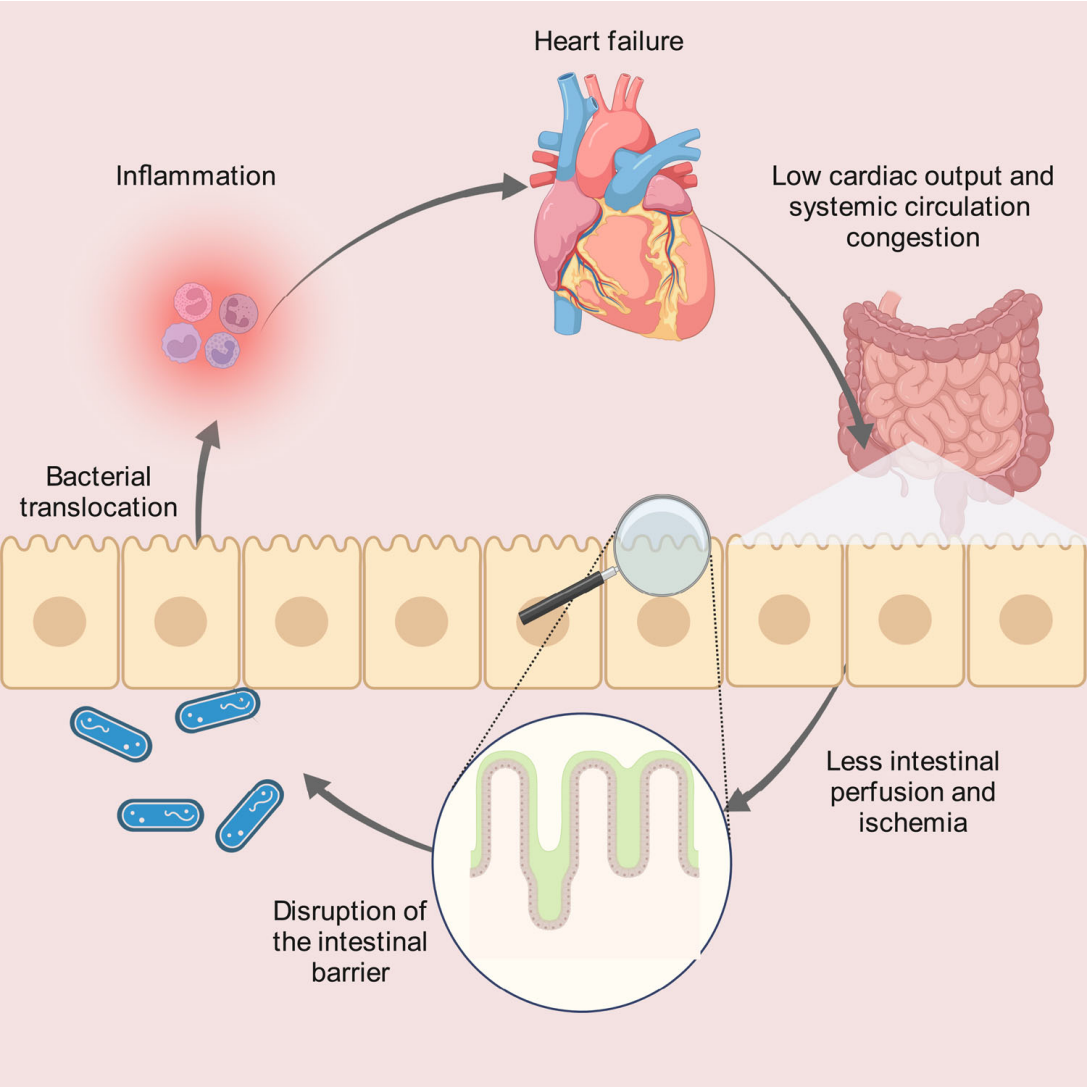
The gut hypothesis of heart failure. The gut hypothesis of HF proposes that low cardiac output and systemic circulation congestion lead to reduced intestinal perfusion, resulting in ischemia and consequently intestinal barrier disruption. A damaged barrier with increased permeability allows bacterial translocation and endotoxins to release into the bloodstream, contributing to inflammation and worsening HF.

Back in 1997, Anker et al. [19] hypothesized that mesenteric venous congestion in CHF leads to an increase in bowel permeability, thus contributing to bacterial translocation and release of endotoxin deteriorating inflammation. In 1999, Anker's team went further, Niebauer et al. [18] proved the hypothesis that there would be an increase in bacterial translocation and endotoxemia caused by altered gut permeability in CHF patients with edema. A damaged barrier can increase permeability, allowing bacterial translocation and endotoxins to enter the bloodstream, contributing to inflammatory responses in HF patients (Figure 1) [18, 20, 28]. This relationship has been further explored and refined to be more concrete. Besides bacterial translocation and endotoxemia, metabolites of the gut microbiota also have an impact on HF. The concept of “refining the gut hypothesis” is first used in a research article indicating the prognostic value of elevated levels of TMAO in HF and suggesting a potential link between the gut microbiota pathway and poor prognosis in HF patients [29]. Besides TMAO, SCFAs, and other metabolites such as TMAVA and phenylacetylgutamine (PAGln) also play an important role in the interaction of the gut microbiota and HF [30, 31, 32]. Up to now, a more specific correlation has been confirmed and the hypothesis has been refined: Congestion in HF would cause increased bowel permeability, followed by bacterial translocation and inflammation, and alterations in the gut microbiota can exacerbate HF through metabolites, mainly TMAO, SFCAs, and resulting in a vicious cycle.

## CHANGES IN GUT MICROBIOTA COMPOSITION

The human gut is colonized by a sophisticated ecosystem of microorganisms, including bacteria, viruses, and fungi, collectively known as the gut microbiota [33]. In this context, we will discuss the changes that occur in the gut microbiota of patients with HF (Table 1). Multiple studies have revealed that the gut microbiota composition is different between HF patients and healthy controls (Table 2) [28, 34, 35, 36, 37, 38, 39, 40]. For instance, certain bacteria, such as *Bacteroides/Prevotellain*, *Eubacterium rectale*, and *Fusobacterium prausnitzii*, are found to be more frequent in HF patients, while others, such as *Coriobacteriaceae*, *Erysipelotrichaceae*, and *Ruminococcaceae*, are decreased [28, 36]. These changes can also impact systemic conditions, including persistent T‐cell activation and increased susceptibility to *Clostridium difficile* infection [40, 41]. Furthermore, the diversity of the gut microbiota is reduced in HF patients.

**Table 1:** Changes of gut microbiota in heart failure patients.

	Increase	Decrease
Phylum	—	*Firmicutes*
Family	*Enterococcaceae*	*Lachnospiraceae, Rumminococcaceae*
Genus	*Bacteroides/Prevotellain*, *Campylobacter*, *Shigella*, *Salmonella*, *Prevotella*, *Hungatella*, *Succinclasticum Enterococcus*, *Synergistete*, *Lactobacillus*	*Blautia*, *Collinsella*, uncl. *Erysipelotrichaceae*, uncl. *Ruminococcaceae*, *Faecalibacterium*, *Ruminococcaceae UCG‐004*, *Ruminococcaceae UCG‐002*, *Lachnospiraceae FCS020* group, *Butyricicoccus*, *Sutterella*, *Lachnospira*, *Ruminiclostridium*
Species	*Eubacterium rectale* a, *Fusobacterium prausnitzii*, *Yersinia enterocolitic*	*Eubacterium rectale* a, *Dorealongicatena*
Fungi	*Candida*, *Candida* species	—

Abbreviation: uncl., unclassified.

^a^ *Eubacterium rectale* is found to increase in HF patients by Sandek et al., while it is also reported to decrease by Kamo et al.

**Table 2:** Summary of studies on changes in the gut microbiota in heart failure.

Source	Time	Sample size^a^	Microbiota	Results	Other
Sandek et al. [28]	2007	22 CHF and 22 controls	*Bacteroides/Prevotellain*, *Eubacterium rectale*, and *Fusobacterium prausnitzii*	Increase	Bacteria were adherent to the mucosa more often
Sandek et al. [34]	2014	21 CHF and 17 control	Both anaerobic and aerobic bacteria	Similar	Bacteria were restricted to the juxtamucosal zone more often
Pasini et al. [35]	2016	60 CHF (NYHA I–II 30, III–IV 30) and 20 controls	Pathogenic bacteria and *Candida* such as *Campylobacter*, *Salmonella*, *Shigella*, Y*ersinia enterocolitica*, and *Candida* species	Increase	Abundancy was different between two NYHA groups
Luedde et al. [36]	2017	20 HFrEF and 20 controls	*Blautia*, *Collinsella*, uncl. *Erysipelotrichaceae* and uncl. *Ruminococcaceae*.	Decrease	Diversity decreased
Kamo et al. [37]	2017	12 HF and 12 controls (age‐matched)	*Eubacterium rectale* and *Dorea longicatena*	Decrease	Older HF patients have less *Bacteroidetes* and more *Proteobacteria*
Kummen et al. [38]	2018	84 stable HFrEF (40 discovery, and 44 validation (NYHA II–IV) and 266 controls	*Genus Prevotella, Hungatella* and *Succinclasticum*	Increase	Bacterial richness decreases in HF patients after adjustment
			*Lachnospiraceae* family,^b^ *Rumminococcaceae Faecalibacterium* and *Bifidobactericeae Bifidobacterium*	Decrease	
Sun et al. [39]	2021	29 Severe CHF (NYHA III–IV) and 30 controls	*Enterococcus* and *Enterococcaceae*	Increase	Lower bacterial richness in chronic HF patients. Remarkable decrease in bacteria generating SCFAs. Increased production of lactic acid.
			Phylum *Firmicutes*, genera *Ruminococcaceae UCG‐002*, *Ruminococcaceae UCG‐004*, *Lachnospiraceae FCS020* group	Decrease	
Huang et al. [40]	2021	30 HFpEF and 30 controls	Phylum *Synergistetes*, genus *Enterococcus* and *Lactobacillus*	Increase	Increase of microbiota linked with inflammation and decrease of microbiota linked with anti‐inflammatory effects
			Genus *Butyricicoccus*, *Sutterella*, *Lachnospira*, and *Ruminiclostridium*	Decrease	

Abbreviations: CHF, chronic heart failure; HF, heart failure; HFpEF, heart failure with preserved ejection fraction; HFrEF, heart failure with reduced ejection fraction; NYHA, New York Heart Association.

^a^ Only includes samples used to identify microbiota changes.

^b^ Includes *Anaerostipes*, *Blautia*, *Coprococcus (3)*, *Fusicatenibacter*, *Lachnospiraceae FCS020*, *NCS2004*, *ND3007*, and *Pseudobutyrivibrio*.

Studies have found that certain bacteria, such as *Bacteroides/Prevotellain*, *Eubacterium rectale*, and *Fusobacterium prausnitzii*, are more frequently found in CHF patients than in controls [28]. Additionally, these bacteria were found to adhere more often to the intestinal mucosa [28]. However, another study by Sandek et al. [34] suggested that more bacteria are restricted to the juxtamucosal zone, after examining both anaerobic and aerobic bacteria in stool. Pasini et al. [35] reported an increase in *Campylobacter*, *Candida*, *Salmonella*, *Shigella*, *Yersinia enterocolitica*, and *Candida* species in the entire CHF group. Specifically, considering colony‐forming units/mL (×10^5^) of stool, *Candida* (37.2 ± 4.4 vs. 2.9 ± 1.1), *Campylobacter* (164.0 ± 6.1 vs. 8.3 ± 1.3), *Shigella* (70.4 ± 17.2 vs. 7.9 ± 1.7), and *Salmonella* (37.6 ± 13.1 vs. 20.2 ± 4.9) were found to be higher in New York Heart Association (NYHA) III to IV than in NYHA I to II CHF patients, while *Yersinia enterocolitica* (24.8 ± 7.5 vs. 23.1 ± 5.9) was similar between the two groups [35]. Elevation of the genera *Enterococcus* and *Enterococcaceae* is also observed in HF patients, leading to an increased lactic acid level [39].

In addition to the increased bacteria in HF patients, certain bacteria are decreased. Studies have shown that the abundance of *Coriobacteriaceae*, *Erysipelotrichaceae*, and *Ruminococcaceae* families is lower in HF patients than in healthy controls when evaluating individual core measurable microbiota (CMM). *Blautia*, *Collinsella*, unclassified *Erysipelotrichaceae*, and unclassified *Ruminococcaceae* also were found less in HF patients [36]. Furthermore, gut microbiota diversity is found to be significantly lower in HF patients [36]. Another study suggested that HF patients have lower levels of *Eubacterium rectale* and *Dorea longicatena* than healthy controls [37]. The trend of *Eubacterium rectale* in HF seems contradictory, with both an increase and decrease in the gut microbiota being reported. The inconsistency may be ascribed to the varieties of the underlying causes for HF. In patients with coronary heart disease, the relative abundance of *Eubacterium rectale* was reported to be remarkably higher compared with the healthy controls [42]. Thus, maybe the abundance of *Eubacterium rectale* is also influenced by the underlying disease that causes HF. Further research is needed for clarifying this contradictory finding. The abundance of Bacteroidetes is higher, while *Proteobacteria* is lower in young compared with older HF patients [37]. The richness of gut microbiota also decreases in HF patients, and they have lower levels of *Lachnospiraceae* family, *Ruminococcaceae Faecalibacterium*, and *Bifidobactericeae Bifidobacterium* [38]. Additionally, phylum *Firmicutes* and bacteria that generate SCFAs are also decreased in the HF group [39].

A direction toward inflammation occurs in changes in gut microbiota, with promoting bacteria flourishing and anti‐inflammatory bacteria diminishing, as confirmed in another study [40]. Moreover, the gut microbiota is more prone to be affected in HF patients, with hospitalized HF patients being more frequently affected by *Clostridium difficile* infection, which is linked with in‐hospital mortality [41]. Not only do changes in gut microbiota affect its composition, but they also impact systemic conditions. Persistent T‐cell activation is shown to be connected with gut microbiota changes in HF patients, which could result in activation of the immune system [40].

Overall, these findings suggest that gut microbiota plays a significant role in the pathophysiology of HF and has the potential of being a therapeutic target.

## CHANGES IN METABOLITES CONTRIBUTING TO HEART FAILURE

**Figure 2:**
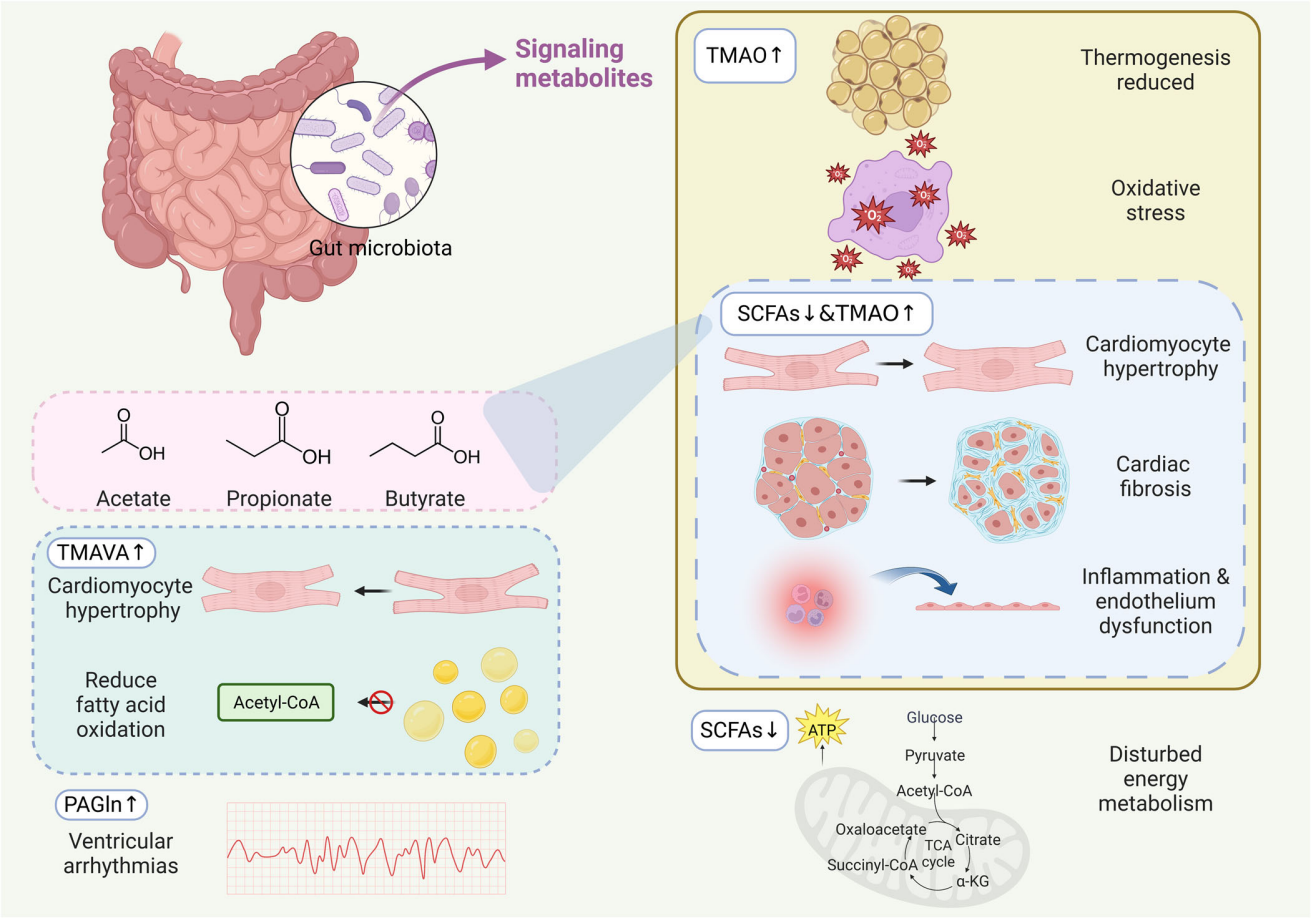
Metabolite changes contribute to HF. TMAO contributes to HF by promoting cardiac fibrosis and hypertrophy, boosting inflammation and endothelial dysfunction, inducing oxidation, and disturbing thermogenesis. A decrease in SCFA levels has been linked to HF, as SCFAs play an important role in preventing cardiac hypertrophy and fibrosis, reducing inflammation, and satisfying energy metabolism. Therefore, maintaining sufficient levels of SCFAs in the body may be beneficial in the prevention and treatment of HF. TMAVA promotes cardiac hypertrophy and reduces fatty acid oxidation. PAGln increases the susceptibility of ventricular arrhythmias in HF. HF, heart failure; PAGln, phenylacetylgutamine; SCFAs, short‐chain fatty acids; TMAO, trimethylamine N‐oxide; TMAVA, *N*,*N*,*N*‐trimethyl‐5‐aminovaleric acid.

It has been discovered that when food is broken down in the gut, it produces trimethylamine N‐oxide (TMAO), SCFAs, and endotoxins under the co‐metabolism between microbiota and host [16]. In individuals with HF, there is an increase in the production of TMAO and endotoxins, which contribute to myocardial fibrosis and hypertrophy. The intestinal barrier is also compromised, leading to inflammation that worsens HF [43]. Furthermore, there is a decrease in the abundance of SCFAs‐producing bacteria, which results in lower levels of SCFAs in HF patients. Other metabolites like TMAVA and PAGln are also involved in HF (Figure 2).

### TMAO

The gut microbiota is involved in the conversion of phosphatidylcholine/choline, l‐carnitine, and betaine from the daily diet into TMA. This TMA is then released into the body circulation, in which it is metabolized into TMAO through oxidization by hepatic flavin monooxygenase (FMO) family members, with FMO3 as the rate‐limiting enzyme [44]. In the metabolism, most TMAO is eliminated by the kidney [45].

Through metabolomics studies, it was reported in 2011 that TMAO, produced by gut microbiota, has the ability to predict CVD risk, showing an elevated risk of cardiovascular events in patients with cardiac diseases taking elective coronary angiography [46]. Since then, further research has been conducted, and TMAO has been found to be associated with multiple CVDs, including HF [43], myocardial infarction [47], hypertension [48], and diabetes [49]. Higher levels of TMAO are confirmed to be associated with a higher long‐term mortality risk [29]. Nine strains have been identified with the capability of generating TMAO in vitro, including two phyla, *Proteobacteria* and *Firmicutes*, and six genera: *Providencia rettgeri*, *Anaerococcus hydrogenalis*, *Clostridium hathewayi*, *Clostridium asparagiforme*, *Clostridium sporogenes*, *Edwardsiella tarda*, *Escherichia fergusonii*, and *Proteus penneri* [50].

As shown in Table 3, TMAO can contribute to HF development at molecular and organ/tissue levels through various and complex interactions, ultimately leading to cardiac fibrosis and hypertrophy, promoting inflammation and endothelial dysfunction, affecting oxidation, and even disrupting thermogenesis.

**Table 3:** Summary of studies about mechanisms of TMAO contributing to HF by time.

Source	Year	Species	Level	Pathway	Effect
Organ et al. [51]	2016	C57BL6/J mice	Organ/system	—	Leads to pulmonary edema, enlargement of heart, increased BNP, decreased left ventricular ejection fraction and myocardial fibrosis
Seldin et al. [52]	2016	Human endothelial cells, LDLR (−/−) mice	Molecule and gene	NF‐κB pathway	Elevated inflammatory gene expression in mice, promotes recruitment of activated leukocytes to endothelial cells
Sun et al. [53]	2016	Human umbilical vein endothelial cells	Molecule	—	Induces inflammation and endothelial dysfunction through ROS‐TXNIP‐NLRP3 inflammasome activation
Chen et al. [54]	2017	Human umbilical vein endothelial cells, aortas from ApoE−/− mice	Molecule	SIRT3–SOD2–mitochondrial ROS signaling pathway (inhibition)	Boosts vascular inflammation through NLRP3 inflammasome activation
Makrecka‐Kuka et al. [55]	2017	ICR mice	Organ/system	—	Impairs β‐oxidation in cardiac mitochondria, promotes cardiac energy metabolism disturbances, and decreases pyruvate metabolism by impairing substrate flux
Li et al. [56]	2019	Sprague‐Dawley rats	Molecule	Smad3 pathway	Promotes myocardial hypertrophy and fibrosis
Brunt et al. [57]	2020	Human and mice	Organ/system	—	Promotes age‐related vascular oxidative stress and endothelial dysfunction
Yoshida et al. [58]	2022	Mice	Molecule	—	Induces decrease of phosphocreatine and ATP levels in heart tissue by suppressing mitochondrial complex IV activity

Abbreviations: ATP, adenosine triphosphate; BNP, brain natriuretic peptide; LVEF, left ventricular ejection fraction.

TMAO can promote cardiac fibrosis and hypertrophy, leading to myocardial damage. Studies have shown that a TMAO‐rich diet can cause pulmonary edema, enlarged heart, lowered left ventricular ejection fraction, and myocardial fibrosis in mice [51]. Moreover, TMAO treatment has been reported to induce cardiac hypertrophy in cardiomyocytes in vitro and promote cardiac hypertrophy and fibrosis in Sprague‐Dawley rats. The level of atrial natriuretic peptide (ANP) and beta‐myosin heavy chain (β‐MHC) also increased, while the size of cardiomyocytes decreased after blocking the Smad3 pathway using a pharmacological inhibitor SIS3 [56].

TMAO exposure triggers an inflammatory response and endothelial dysfunction. TMAO is closely related to inflammatory status and increased inflammatory gene expression has been observed in mice. TMAO has been reported to increase inflammation in peritoneal dialysis patients [59]. To be more specific, the production of P‐selectin induced by tumor necrosis factor‐alpha (TNF‐α) was found to increase in mesothelial cells by TMAO and TMAO promoted TNF‐α induced by high glucose and expression of CCL2 in endothelial cells [59]. In addition, activated leukocytes are recruited to endothelial cells through the NF‐κB pathway in mice [52]. TMAO has also been found to induce both endothelial dysfunction and inflammation by activating the ROS–TXNIP–NLRP3 inflammasome [53]. Furthermore, TMAO activates the NLRP3 inflammasome by inhibition of the SIRT3–SOD2–mitochondrial ROS signaling pathway [54].

Furthermore, TMAO alters the oxidation process, leading to disturbances in energy metabolism. In both mice and healthy individuals, TMAO accelerates age‐related vascular oxidative stress and endothelial dysfunction. This is evidenced by the associations between TMAO and higher nitrotyrosine abundance in endothelial cells after biopsy, as well as oxidative stress‐related dysfunction of endothelium [57]. Additionally, TMAO could impair β‐oxidation in cardiac mitochondria, promote cardiac energy metabolism disturbances, and decrease pyruvate metabolism by impairing substrate flux, according to another study [55].

Interestingly, TMAO can also affect thermogenesis, which in turn may promote HF. Brown adipose tissue (BAT) is known for its thermogenic properties, but it also performs other functions. Metabolomic analysis has shown that elevated plasma TMAO levels are related to reduced BAT thermogenesis [58]. Experiments on mice have also revealed that TMAO can decrease phosphocreatine and adenosine triphosphate (ATP) levels in heart tissue by suppressing activity of mitochondrial complex IV [58]. Moreover, patients with dilated cardiomyopathy have been found to have elevated TMAO levels and low body temperature, which is associated with poor prognosis of HF. This suggests that TMAO may cause dysfunction of BAT, thus promoting HF [58].

### SCFA

**Figure 3:**
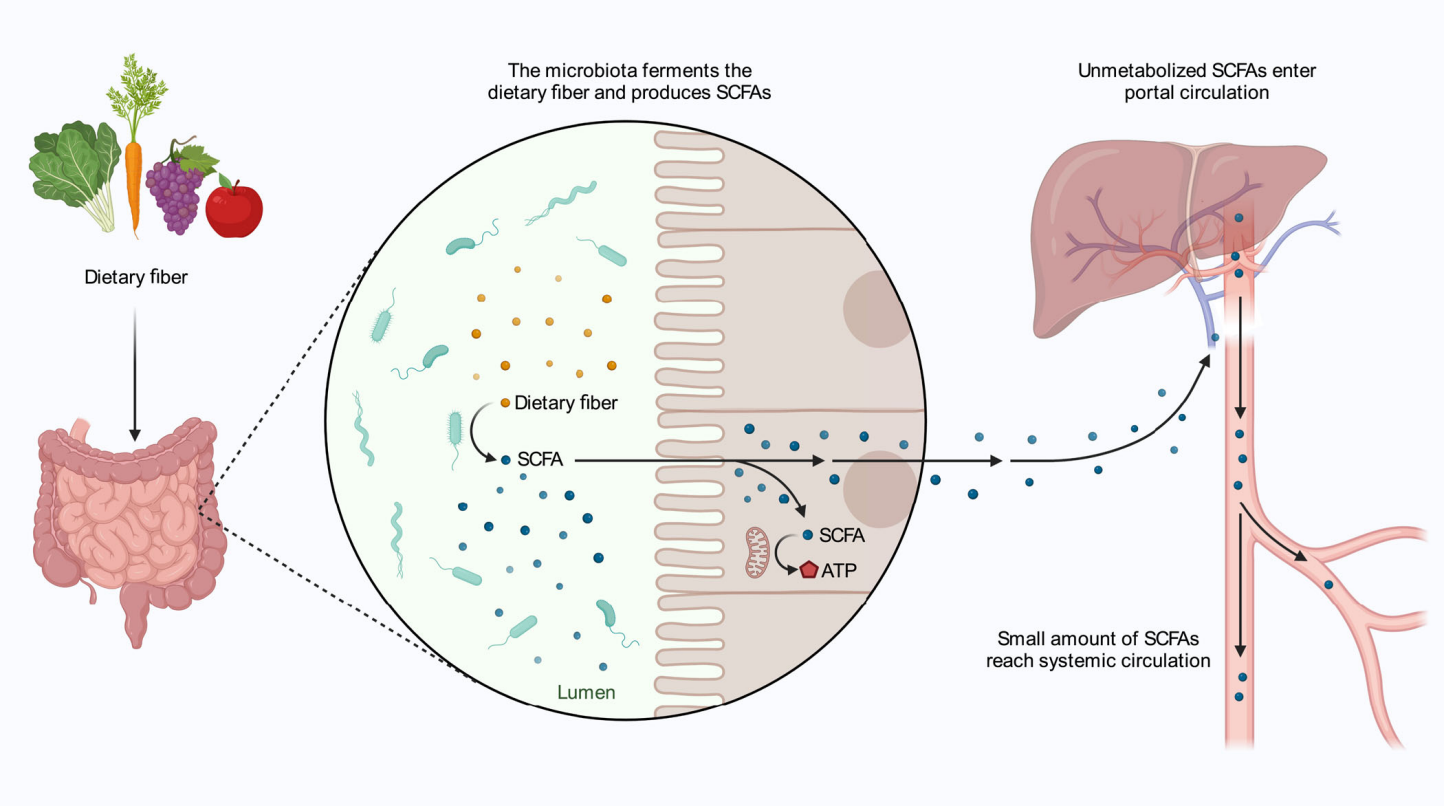
Metabolism of short‐chain fatty acids (SCFAs). SCFAs are mainly produced from dietary fiber by the gut microbiota and used as an energy source by the mitochondria. Unmetabolized SCFAs enter portal circulation, and a small amount of SCFAs reach systemic circulation.

SCFAs are saturated aliphatic organic acids composed of 1 to 6 carbon atoms, with acetate (C2), propionate (C3), and butyrate (C4) being the most abundant (≥95%) [60, 61]. SCFAs are mainly produced from dietary fiber by the gut microbiota. Unmetabolized SCFAs would enter portal circulation, with a small number of SCFAs reaching the systemic circulation (Figure 3). In failing hearts, energy starvation occurs due to impaired oxidation of long‐chain fatty acids caused by reduced activity of carnitine palmitoyltransferase 1 (CPT1) on the outer mitochondrial membrane. However, studies have reported that SCFAs can bypass CPT1 and provide energy to the failing heart [24]. SCFAs are transported into colonocytes by monocarboxylate transporters (MCT), with MCT1 being the most widely distributed subtype, in addition to passive distribution [30].

Studies have shown a decrease in SCFA‐producing bacteria in hypertensive HF rats, which is also observed in CHF patients [39, 62]. Subsequent research into SCFA subtypes has revealed lower levels of plasma propionate, butyrate, and isovalerate in HF patients, while no difference is observed in acetate and valerate levels [63]. Interestingly, in patients with congestive HF, higher levels of SCFAs, especially propionate and butyrate, are associated with better cardiac function [30].

SCFAs play a crucial role in the regulation of proliferation, differentiation, and functions of intestinal epithelial cells (IECs) [64]. As the primary energy source of IECs, butyrate consumes up to 3/4 of oxygen for human colonocytes and the converted into ketone bodies [64]. Besides providing energy for healthy cells, butyrate inhibits the expansion of cancerous cells, which is known as the Warburg effect or butyrate paradox [65]. The junctional integrity of IECs could also be promoted by butyrate [66]. Additionally, SFCA can prevent pathophysiological changes in the heart, including cardiac fibrosis, inflammation, and energy metabolism disturbances.

In both mouse models of hypertensive cardiac damage and atherosclerosis, propionate has been found to mitigate cardiac fibrosis, hypertrophy, and vascular dysfunction. Additionally, SCFAs have been linked with an immune‐regulatory role, as propionate has been shown to attenuate systemic inflammation with fewer effector memory T cells and T helper 17 cells in the spleen, and less severe local infiltration of cardiac immune cells [67]. In fiber‐depleted mice, SCFAs have been found to have protective effects on cardiac hypertrophy and fibrosis, which are mediated by SCFAs receptors G‐protein‐coupled receptors (GPCR) 43/GPCR109A, and regulated by the level of L‐3,4‐dihydroxyphenylalanine and DNA‐methylation modulated regulatory T cells [68].

Inflammation is closely related to endothelial cells, and these cells cooperate with immune cells in regulating inflammation, which could be activated by lipopolysaccharide (LPS) and TNF‐α [69]. Once activated, endothelial cells can strengthen the inflammatory response [70]. LPS is a major unit of the Gram‐negative bacteria's cell wall and could induce inflammation through several signaling pathways, which can be inhibited by SCFAs via GPCRs and histone deacetylases (HDACs) [71]. In general, SCFAs can downregulate pro‐inflammatory cytokines and up‐regulate anti‐inflammatory cytokines. For instance, by activating free fatty acid receptors, acetate can reduce the secretion of TNF‐α from mononuclear cells [72]. Butyrate and propionate can downregulate TNF expression and nitric oxide synthase (NOS) in neutrophils induced by LPS [73]. SCFAs also inhibit the generation of other pro‐inflammatory factors, such as IL‐6, IL‐8, and MCP‐1 [74, 75], and induce the release of IL‐10, which functions as an anti‐inflammatory factor, in monocytes [76]. It is suggested that SCFAs perform their anti‐inflammatory effects on LPS‐ or TNF‐α‐stimulated endothelial cells by activating GPCRs 41/43 and inhibiting HDACs [77]. Apart from SCFAs’ effect in modulating inflammation, SCFAs can interact with endothelial cells directly, and their production could improve vascular endothelial function [78].

SCFAs play a significant role in energy metabolism, accounting for about 10% of daily energy demand [79]. For colonic epithelium, SCFAs are the primary energy resource, contributing to approximately 75% of energy metabolism [80]. Acetate serves as a precursor for hepatic and adipocyte lipogenesis, while butyrate is associated with cell growth, differentiation, and mitochondrial activity, improves insulin sensitivity, prevents obesity induced by diet without causing hypophagia, and enhances intestinal barrier function [81, 82]. Propionate, on the other hand, is a necessary substrate for gluconeogenesis and has been shown to reduce food intake and cholesterol synthesis [81, 83]. SCFAs have been reported to support the failing heart since they can bypass CPT1 and be used as an energy source [24]. In summary, SCFAs’ metabolic rate is essential to energy balance, and they play critical roles in various metabolic processes.

### Other agents

Change in gut microbiota is a complex process that interacts with HF in various ways. Besides TMAO and SCFAs, the gut microbiota also impacts HF through other agents, including TMAVA, PAGln, and other molecular actors. TMAVA is produced by the gut microbiota from trimethyllysine and has been found to be elevated and associated with an increased risk of cardiac mortality and transplantation [31]. Studies have shown that TMAVA can reduce fatty acid oxidation and promote cardiac hypertrophy in mouse models [31]. PAGln is generated by the gut microbiota and the human liver and has been identified as a risk factor and prognostic indicator of HF [84, 85]. It is related to the presence and severity of HF both clinically and mechanistically [32]. In an HF mouse model, PAGln increased the chance of ventricular arrhythmias by TLR4/AKT/mTOR signaling pathway activation [86].

## GUT MICROBIOTA AND HEART FAILURE INTERVENTIONS/TREATMENTS

**Figure 4:**
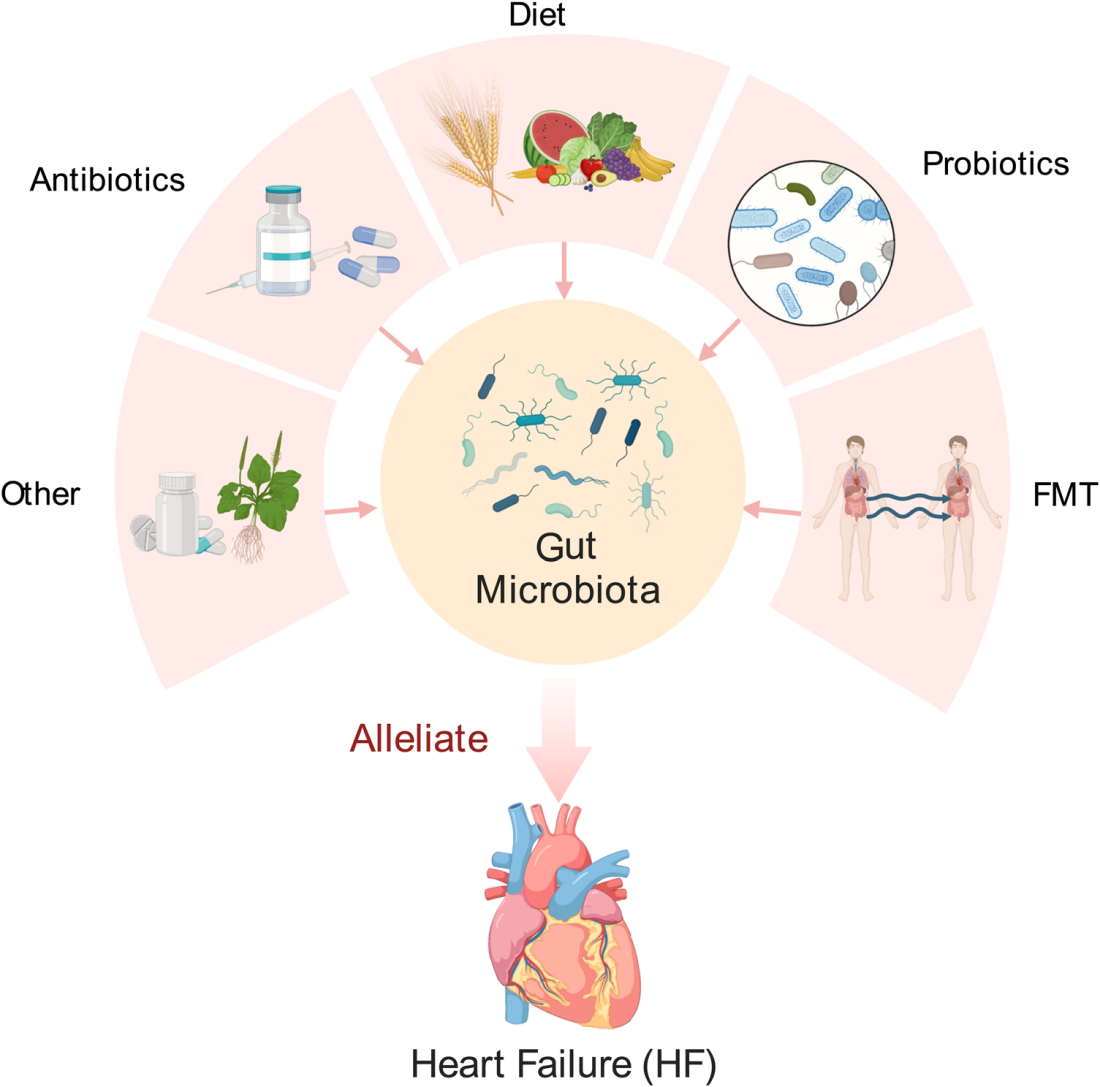
Intervention on the gut microbiota in HF. Interventions targeting the gut microbiota in HF include dietary interventions, such as the Dietary Approaches to Stop Hypertension (DASH) and Mediterranean diets, probiotic therapy, fecal microbiota transplantation, antibiotics, and other potential approaches.

Recent research has highlighted the importance of gut microbiota in the development and progress of HF. This has led to the exploration of several dietary interventions, including the Dietary Approaches to Stop Hypertension (DASH) diet and Mediterranean diet, probiotic therapy, fecal microbiota transplantation, and antibiotics, as potential treatments for HF (Figure 4). In addition, other interventions, such as vitamin D, B vitamins, berberine, and 3,3‐dimethyl‐1‐butanol (DMB), have been investigated for their potential to reduce HF risk by targeting the gut microbiota. In this section, we will discuss findings regarding these interventions and their effects on the gut microbiota in the context of HF.

### Dietary interventions

Gut microbiota is closely related to daily diets, and even a short‐term adjustment of the diet, for only 5 days, is sufficient to alter the composition of gut microbiota and induce corresponding changes for adaptation [87]. As the name suggests, the DASH diet was designed for stopping hypertension through dietary approaches, and it has been regarded as an effective dietary intervention in lowering blood pressure, especially with reduced dietary sodium [88]. Compared with the daily diet, the DASH diet is richer in vegetables, fruits, and low‐fat dairy products [89]. In a cohort study involving 35,004 participants with a median follow‐up period of 22 years, it was suggested that the DASH diet could lower the risk of HF [90]. In HF patients, the DASH diet can also improve 6‐min walking test performance, compliance of artery, exercise capacity, and quality of life scores evaluated after an intervention for 3 months [91]. The Mediterranean diet describes the shared diet pattern among at least 16 countries bordering the Mediterranean Sea [92]. Besides being rich in fruits and vegetables, which is similar to that of the DASH diet, the Mediterranean diet is also characterized by bread, cereals of other forms, potatoes, beans, nuts, seeds, olive oil, little red meat, and low to moderate amounts of dairy products, fish, poultry and wine [92]. Although the Mediterranean diet was linked with lower all‐cause mortality in CVD patients, a pre‐specified secondary analysis from the PREvención con DIeta MEDiterránea (PREDIMED) trial did not find a significant decrease in HF incidence [93, 94]. However, further trials are needed to explore the effects of the Mediterranean diet on HF patients, as this analysis may not be powerful enough to provide solid conclusions [94]. In general, the DASH diet and the Mediterranean diet may assist in the prevention of HF, but high‐quality evidence is needed to establish their efficacy [95].

### Probiotic therapy

The definition of a probiotic by the Food and Agriculture Organization of the United Nations and the World Health Organization (FAO/WHO) has been widely adopted, which describes a probiotic as “live microorganisms that, when administered in adequate amounts, confer a health benefit on the host” [96]. Pieces of literature have revealed that *Lactobacillus rhamnosus GR‐1* can significantly attenuate hypertrophy and improve not only the systolic but also the diastolic function of the left ventricle, preserve LVEF and fractional shortening after a 6‐week follow‐up, indicating that probiotic therapy has the potential to attenuate HF [97]. In CHF patients, a multistrain probiotic has been reported to reduce sarcopenia and improve functional capacity by regulating the Wnt signaling pathway [98]. A randomized, double‐blind, placebo‐controlled pilot trial targeting HF patients using *Saccharomyces boulardii* for 3 months showed that it could improve LVEF, shorten left atrial diameter, and lower total cholesterol and uric acid levels [99]. Another randomized, triple‐blind, controlled trial suggested that probiotic yogurt might be helpful in relieving the inflammatory status in CHF patients by elevating sTWEAK levels [100]. However, the randomized Targeting Gut Microbiota to Treat Heart Failure (GutHeart) trial found that treatment with *Saccharomyces boulardii* or rifaximin for 3 months, on top of standard of care, had no significant effect on LVEF, diversity of microbiota, or the measured biomarkers in HFrEF patients [101]. Further research and studies are needed to figure out the potential effects and underlying mechanisms of probiotic therapy in HF.

### Fecal microbiota transplantation

The procedure of transplanting stools from a healthy donor into another patient's intestine is known as fecal microbiota transplantation (FMT) or stool transplantation [102]. FMT has primarily been used to treat recurrent *Clostridium difficile* infection [102, 103]. However, studies have also focused on the potential of FMT in treating chronic diseases, and the super‐donor phenomenon has been observed, indicating that FMT may be more successful when using feces from specific donors [104]. Nonetheless, FMT can also carry risks, such as importing viral communities together with the necessary microbiota [105]. While the potential effects of FMT on HF are not well studied, it is important to consider both the benefits and risks associated with the procedure, and FMT may hold promise as a supplementary treatment for HF.

### Antibiotics

The misuse of antibiotics can disrupt an individual's microbiota and cause harmful effects. However, in some cases, antibiotics may be helpful since microbial translocation can cause harmful events. For example, after an ST‐elevation myocardial infarction, microbial translocation can induce inflammation and cardiovascular events, which can be alleviated by antibiotics [106]. Rifaximin is also widely used to treat microbiota toxicity and translocation by performing anti‐inflammatory effects and promoting the growth of *bifidobacteria* and *lactobacillus* [107, 108]. Unfortunately, the effects of antibiotics on gut microbiota in HF have not been extensively studied. It is important to remember that antibiotics are a double‐edged sword, with potential benefits and risks that need to be carefully weighed.

### Other interventions

Evidence implies that high TMAO levels are linked with a deficiency in vitamin D, indicating that vitamin D may help reduce TMAO levels in patients [109]. Moreover, a study has proposed that B vitamins + vitamin D can cause changes in choline metabolism, resulting in further lowering of TMAO levels when compared to vitamin D alone [110]. In addition, oral intake of berberine for 4 months has been shown to decrease TMAO production in animal intestines and lower TMA and TMAO levels in both the feces and plasma of patients through vitamin‐like effects [44]. Furthermore, DMB has been reported to raise cardiac function and alleviate cardiac remodeling in HF mice induced by pressure overload by downregulating plasma TMAO levels, which inhibits the TGF‐β1/Smad3 and p65 NF‐κB signaling pathway and attenuates cardiac hypertrophy, fibrosis, and inflammation [111]. Additionally, traditional Chinese medicine (TCM) has been shown to interact with gut microbiota, as TCM regulates metabolism and is metabolized by gut microbiota [112].

## CONCLUSION

The gut hypothesis of HF highlights the potential of targeting gut microbiota for the interventions or treatments of HF. The composition and diversity of gut microbiota are altered in HF, and it produces more TMAO and fewer SCFAs compared to healthy individuals. TMAO promotes HF by promoting cardiac hypertrophy, fibrosis, inflammation, and endothelial dysfunction, while SCFAs have a protective role by preventing pathophysiological changes and satisfying energy metabolism. Other microbiota metabolites like TMAVA and PAGln may also play a role in HF. Dietary interventions and probiotic therapy have shown potential in attenuating HF and improving cardiac function. However, further research and studies are needed to determine the effectiveness of FMT and antibiotics in HF treatment. Overall, the gut microbiota represents a promising avenue for the development of novel HF treatments.

## AUTHOR CONTRIBUTIONS

An‐Tian Chen wrote the manuscript. Jian Zhang and Yuhui Zhang supervised this project. All authors have read the final manuscript and approved it for publication.

## CONFLICT OF INTEREST STATEMENT

The authors declare no conflict of interest.

## Data Availability

This manuscript does not generate any code or data. Supplementary materials (graphical abstract, slides, videos, Chinese translated version and update materials) may be found in the online DOI or iMeta Science http://www.imeta.science/.

## References

[1] de Vos, Willem M. , Herbert Tilg , Matthias Van Hul , and Patrice D. Cani . 2022. “Gut Microbiome and Health: Mechanistic Insights.” Gut 71: 1020–32. 10.1136/gutjnl-2021-326789 35105664 PMC8995832

[2] James, Spencer L. , Degu Abate , Kalkidan Hassen Abate , Solomon M. Abay , Cristiana Abbafati , Nooshin Abbasi , Hedayat Abbastabar , et al. 2018. “Global, Regional, and National Incidence, Prevalence, and Years Lived With Disability for 354 Diseases and Injuries for 195 Countries and Territories, 1990–2017: A Systematic Analysis for the Global Burden of Disease Study 2017.” The Lancet 392: 1789–858. 10.1016/s0140-6736(18)32279-7 PMC6227754 30496104

[3] Groenewegen, Amy , Frans H. Rutten , Arend Mosterd , and Arno W. Hoes . 2020. “Epidemiology of Heart Failure.” European Journal of Heart Failure 22: 1342–56. 10.1002/ejhf.1858 32483830 PMC7540043

[4] Denolin, H. , H. Kuhn , H. Krayenbuehl , F. Loogen , and A. Reale . 1983. “The Defintion of Heart Failure.” European Heart Journal 4: 445–8. 10.1093/oxfordjournals.eurheartj.a061500 6628420

[5] Heidenreich, Paul A. , Biykem Bozkurt , David Aguilar , Larry A. Allen , Joni J. Byun , Monica M. Colvin , Anita Deswal , et al. 2022. “2022 AHA/ACC/HFSA Guideline for the Management of Heart Failure: A Report of the American College of Cardiology/American Heart Association Joint Committee on Clinical Practice Guidelines.” Circulation 145: e895–1032. 10.1161/CIR.0000000000001063 35363499

[6] Tang, W. H. Wilson , Daniel Y. Li , and Stanley L. Hazen . 2019. “Dietary Metabolism, the Gut Microbiome, and Heart Failure.” Nature Reviews Cardiology 16: 137–54. 10.1038/s41569-018-0108-7 30410105 PMC6377322

[7] Gustafsson, Finn , and Joseph G. Rogers . 2017. “Left Ventricular Assist Device Therapy in Advanced Heart Failure: Patient Selection and Outcomes.” European Journal of Heart Failure 19: 595–602. 10.1002/ejhf.779 28198133

[8] Chambers, Daniel C. , Michael Perch , Andreas Zuckermann , Wida S. Cherikh , Michael O. Harhay , Don Hayes , Eileen Hsich , et al. 2021. “The International Thoracic Organ Transplant Registry of the International Society for Heart and Lung Transplantation: Thirty‐Eighth Adult Lung Transplantation Report—2021; Focus on Recipient Characteristics.” The Journal of Heart and Lung Transplantation 40: 1060–72. 10.1016/j.healun.2021.07.021 34446355 PMC10285650

[9] Ley, Ruth E. , Daniel A. Peterson , and Jeffrey I. Gordon . 2006. “Ecological and Evolutionary Forces Shaping Microbial Diversity in the Human Intestine.” Cell 124: 837–48. 10.1016/j.cell.2006.02.017 16497592

[10] Dogra, Shaillay Kumar , Joel Doré , and Sami Damak . 2020“Gut Microbiota Resilience: Definition, Link to Health and Strategies for Intervention.” Frontiers in Microbiology 11: 572921. 10.3389/fmicb.2020.572921 33042082 PMC7522446

[11] Qin, Junjie , Ruiqiang Li , Jeroen Raes , Manimozhiyan Arumugam , Kristoffer Solvsten Burgdorf , Chaysavanh Manichanh , Trine Nielsen , et al. 2010. “A Human Gut Microbial Gene Catalogue Established ny Metagenomic Sequencing.” Nature 464: 59–65. 10.1038/nature08821 20203603 PMC3779803

[12] Sharon, Gil , Nikki Jamie Cruz , Dae‐Wook Kang , Michael J. Gandal , Bo Wang , Young‐Mo Kim , Erika M. Zink , et al. 2019. “Human Gut Microbiota From Autism Spectrum Disorder Promote Behavioral Symptoms in Mice.” Cell 177: 1600–18.e17. 10.1016/j.cell.2019.05.004 31150625 PMC6993574

[13] Hu, Xu , Tao Wang , and Feng Jin . 2016. “Alzheimer's Disease and Gut Microbiota.” Science China Life Sciences 59: 1006–23. 10.1007/s11427-016-5083-9 27566465

[14] Quigley, Eamonn M. M . 2017. “Microbiota‐Brain‐Gut Axis and Neurodegenerative Diseases.” Current Neurology and Neuroscience Reports 17: 94. 10.1007/s11910-017-0802-6 29039142

[15] Jiang, Shuaiming , Denghui Chen , Chenchen Ma , Huanwei Liu , Shi Huang , and Jiachao Zhang . 2022. “Establishing a Novel Inflammatory Bowel Disease Prediction Model Based on Gene Markers Identified From Single Nucleotide Variants of the Intestinal Microbiota.” iMeta 1: e40. 10.1002/imt2.40 PMC10989788 38868717

[16] Rosa, Giulio La , and Luigi Marzio Biasucci . 2016. “The Gut Microbiota and Atherosclerosis: the State Of the Art and Novel Perspectives.” Cardiovascular Innovations and Applications 1: 433–42. 10.15212/CVIA.2016.0027

[17] Fan, Yong , Jiajun Ying , Hongchuang Ma , and Hanbin Cui . 2023. “Microbiota‐Related Metabolites Fueling the Understanding of Ischemic Heart Disease.” iMeta 2: e94. 10.1002/imt2.94 PMC10989774 38868424

[18] Niebauer, Josef , Hans‐Dieter Volk , Michael Kemp , Martin Dominguez , Ralf R. Schumann , Mathias Rauchhaus , Philip A. Poole‐Wilson , Andrew J. S. Coats , and Stefan D. Anker . 1999. “Endotoxin and Immune Activation in Chronic Heart Failure: a Prospective Cohort Study.” The Lancet 353: 1838–42. 10.1016/s0140-6736(98)09286-1 10359409

[19] Anker, Stefan D. , Karl R. Egerer , Hans‐Dieter Volk , Wolfgang J. Kox , Philip A. Poole‐Wilson , and Andrew J. S. Coats . 1997. “Elevated Soluble CD14 Receptors and Altered Cytokines in Chronic Heart Failure.” The American Journal of Cardiology 79: 1426–30. 10.1016/s0002-9149(97)00159-8 9165177

[20] Nagatomo, Yuji , and W. H. Wilson Tang . 2015. “Intersections Between Microbiome and Heart Failure: Revisiting the Gut Hypothesis.” Journal of Cardiac Failure 21: 973–80. 10.1016/j.cardfail.2015.09.017 26435097 PMC4666782

[21] Rodrigues, Alexandre , Alexandre Gonçalves , Juliana Morais , Ricardo Araujo , and Inês Falcão‐Pires . 2023. “Diet‐Induced Microbiome's Impact on Heart Failure: A Double‐Edged Sword.” Nutrients 15: 1223. 10.3390/nu15051223 36904222 PMC10004801

[22] Guan, Xueqing , and Zhijun Sun . 2023. “The Role of Intestinal Flora and its Metabolites in Heart Failure.” Infection and Drug Resistance 16: 51–64. 10.2147/idr.S390582 36636378 PMC9830706

[23] Mamic, Petra , Michael Snyder , and W. H. Wilson Tang . 2023. “Gut Microbiome‐Based Management of Patients with Heart Failure.” Journal of the American College of Cardiology 81: 1729–39. 10.1016/j.jacc.2023.02.045 37100490

[24] Carley, Andrew N. , Santosh K. Maurya , Matthew Fasano , Yang Wang , Craig H. Selzman , Stavros G. Drakos , and E. Douglas Lewandowski . 2021. “Short‐Chain Fatty Acids Outpace Ketone Oxidation in the Failing Heart.” Circulation 143: 1797–808. 10.1161/circulationaha.120.052671 33601938 PMC8096711

[25] Groschwitz, Katherine R. , and Simon P. Hogan . 2009. “Intestinal Barrier Function: Molecular Regulation and Disease Pathogenesis.” Journal of Allergy and Clinical Immunology 124: 3–20. quiz 21‐22. 10.1016/j.jaci.2009.05.038 19560575 PMC4266989

[26] Mitsudo, Sumi , and Lawrence J. Brandt . 1992. “Pathology of Intestinal Ischemia.” Surgical Clinics of North America 72: 43–63. 10.1016/s0039-6109(16)45627-6 1731389

[27] Chang, Marisol , Erik B. Kistler , and Geert W. Schmid‐Schönbein . 2012. “Disruption of the Mucosal Barrier During Gut Ischemia Allows Entry of Digestive Enzymes into the Intestinal Wall.” Shock 37: 297–305. 10.1097/SHK.0b013e318240b59b 22089198 PMC3288241

[28] Sandek, Anja , Juergen Bauditz , Alexander Swidsinski , Sabine Buhner , Jutta Weber‐Eibel , Stephan von Haehling , Wieland Schroedl , et al. 2007. “Altered Intestinal Function in Patients with Chronic Heart Failure.” Journal of the American College of Cardiology 50: 1561–9. 10.1016/j.jacc.2007.07.016 17936155

[29] Tang, W. H. Wilson , Zeneng Wang , Yiying Fan , Bruce Levison , Jennie E. Hazen , Lillian M. Donahue , Yuping Wu , and Stanley L. Hazen . 2014. “Prognostic Value of Elevated Levels of Intestinal Microbe‐Generated Metabolite Trimethylamine‐N‐Oxide in Patients with Heart Failure: Refining the Gut Hypothesis.” Journal of the American College of Cardiology 64: 1908–14. 10.1016/j.jacc.2014.02.617 25444145 PMC4254529

[30] Hu, Tongtong , Qingqing Wu , Qi Yao , Kebing Jiang , Jiabin Yu , and Qizhu Tang . 2022. “Short‐Chain Fatty Acid Metabolism and Multiple Effects on Cardiovascular Diseases.” Ageing Research Reviews 81: 101706. 10.1016/j.arr.2022.101706 35932976

[31] Zhao, Mingming , Haoran Wei , Chenze Li , Rui Zhan , Changjie Liu , Jianing Gao , Yaodong Yi , et al. 2022. “Gut Microbiota Production of trimethyl‐5‐aminovaleric Acid Reduces Fatty Acid Oxidation and Accelerates Cardiac Hypertrophy.” Nature Communications 13: 1757. 10.1038/s41467-022-29060-7 PMC8976029 35365608

[32] Romano, Kymberleigh A. , Ina Nemet , Prasenjit Prasad Saha , Arash Haghikia , Xinmin S. Li , Maradumane L. Mohan , Beth Lovano , et al. 2023. “Gut Microbiota‐Generated Phenylacetylglutamine and Heart Failure.” Circulation: Heart Failure 16: e009972. 10.1161/CIRCHEARTFAILURE.122.009972 36524472 PMC9851997

[33] Sorboni, Shokufeh Ghasemian , Hanieh Shakeri Moghaddam , Reza Jafarzadeh‐Esfehani , and Saman Soleimanpour . 2022. “A Comprehensive Review on the Role of the Gut Microbiome in Human Neurological Disorders.” Clinical Microbiology Reviews 35: e0033820. 10.1128/cmr.00338-20 34985325 PMC8729913

[34] Sandek, Anja , Alexander Swidsinski , Wieland Schroedl , Alastair Watson , Miroslava Valentova , Ralph Herrmann , Nadja Scherbakov , et al. 2014. “Intestinal Blood Flow in Patients with Chronic Heart Failure.” Journal of the American College of Cardiology 64: 1092–102. 10.1016/j.jacc.2014.06.1179 25212642

[35] Pasini, Evasio , Roberto Aquilani , Cristian Testa , Paola Baiardi , Stefania Angioletti , Federica Boschi , Manuela Verri , and Francesco Dioguardi . 2016. “Pathogenic Gut Flora in Patients with Chronic Heart Failure.” JACC: Heart Failure 4: 220–7. 10.1016/j.jchf.2015.10.009 26682791

[36] Luedde, Mark , Thorben Winkler , Femke‐Anouska Heinsen , Malte C. Rühlemann , Martina E. Spehlmann , Amer Bajrovic , Wolfgang Lieb , et al. 2017. “Heart Failure is Associated with Depletion of Core Intestinal Microbiota.” ESC Heart Failure 4: 282–90. 10.1002/ehf2.12155 28772054 PMC5542738

[37] Kamo, Takehiro , Hiroshi Akazawa , Wataru Suda , Akiko Saga‐Kamo , Yu Shimizu , Hiroki Yagi , Qing Liu , et al. 2017. “Dysbiosis and Compositional Alterations with Aging in the Gut Microbiota of Patients with Heart Failure.” PLOS ONE 12: e0174099. 10.1371/journal.pone.0174099 28328981 PMC5362204

[38] Kummen, Martin , Cristiane C. K. Mayerhofer , Beate Vestad , Kaspar Broch , Ayodeji Awoyemi , Christopher Storm‐Larsen , Thor Ueland , et al. 2018. “Gut Microbiota Signature in Heart Failure Defined From Profiling of 2 Independent Cohorts.” Journal of the American College of Cardiology 71: 1184–6. 10.1016/j.jacc.2017.12.057 29519360

[39] Sun, Weiju , Debing Du , Tongze Fu , Ying Han , Peng Li , and Hong Ju . 2022. “Alterations of the Gut Microbiota in Patients With Severe Chronic Heart Failure.” Frontiers in Microbiology 12: 813289. 10.3389/fmicb.2021.813289 35173696 PMC8843083

[40] Huang, Ziyin , Xiaofei Mei , Yufeng Jiang , Tan Chen , and Yafeng Zhou . 2022. “Gut Microbiota in Heart Failure Patients With Preserved Ejection Fraction (GUMPTION Study).” Frontiers in Cardiovascular Medicine 8: 803744. 10.3389/fcvm.2021.803744 35071367 PMC8770938

[41] Mamic, Petra , Paul A. Heidenreich , Haley Hedlin , Lakshika Tennakoon , and Kristan L. Staudenmayer . 2016. “Hospitalized Patients with Heart Failure and Common Bacterial Infections: A Nationwide Analysis of Concomitant *Clostridium Difficile* Infection Rates and In‐Hospital Mortality.” Journal of Cardiac Failure 22: 891–900. 10.1016/j.cardfail.2016.06.005 27317844

[42] Li, Wenlong , Huijun Li , Shaolan Wang , Keyang Han , Yuan Liu , Zhen An , Hui Wu , et al. 2022. “Regional Pattern and Signatures of Gut Microbiota in Rural Residents with Coronary Heart Disease: A Metagenomic Analysis.” Frontiers in Cellular and Infection Microbiology 12: 1007161. 10.3389/fcimb.2022.1007161 36519129 PMC9742380

[43] Jia, Qiujin , Lirong Wang , Xiaonan Zhang , Yuejia Ding , Hao Li , Yingxi Yang , Ao Zhang , et al. 2020. “Prevention and Treatment of Chronic Heart Failure Through Traditional Chinese Medicine: Role of the Gut Microbiota.” Pharmacological Research 151: 104552. 10.1016/j.phrs.2019.104552 31747557

[44] Ma, Shu‐Rong , Qian Tong , Yuan Lin , Li‐Bin Pan , Jie Fu , Ran Peng , Xian‐Feng Zhang , et al. 2022. “Berberine Treats Atherosclerosis Via A Vitamine‐Like Effect Down‐Regulating Choline‐TMA‐TMAO Production Pathway in Gut Microbiota.” Signal Transduction and Targeted Therapy 7: 207. 10.1038/s41392-022-01027-6 35794102 PMC9259588

[45] Canyelles, Marina , Carla Borràs , Noemí Rotllan , Mireia Tondo , Joan Carles Escolà‐Gil , and Francisco Blanco‐Vaca . 2023. “Gut Microbiota‐Derived TMAO: A Causal Factor Promoting Atherosclerotic Cardiovascular Disease? International Journal of Molecular Sciences 24: 1940. 10.3390/ijms24031940 36768264 PMC9916030

[46] Wang, Zeneng , Elizabeth Klipfell , Brian J. Bennett , Robert Koeth , Bruce S. Levison , Brandon DuGar , Ariel E. Feldstein , et al. 2011. “Gut Flora Metabolism of Phosphatidylcholine Promotes Cardiovascular Disease.” Nature 472: 57–63. 10.1038/nature09922 21475195 PMC3086762

[47] Tan, Yu , Zhaoxue Sheng , Peng Zhou , Chen Liu , Hanjun Zhao , Li Song , Jiannan Li , et al. 2019. “Plasma Trimethylamine N‐Oxide as a Novel Biomarker for Plaque Rupture in Patients with ST‐Segment‐Elevation Myocardial Infarction.” Circulation: Cardiovascular Interventions 12: e007281. 10.1161/CIRCINTERVENTIONS.118.007281 30599768

[48] Jiang, Shan , Yongjie Shui , Yu Cui , Chun Tang , Xiaohua Wang , Xingyu Qiu , Weipeng Hu , et al. 2021. “Gut Microbiota Dependent Trimethylamine N‐Oxide Aggravates Angiotensin II‐induced Hypertension.” Redox Biology 46: 102115. 10.1016/j.redox.2021.102115 34474396 PMC8408632

[49] Li, Doudou , Ying Lu , Shuai Yuan , Xiaxia Cai , Yuan He , Jie Chen , Qiong Wu , et al. 2022. “Gut Microbiota‐Derived Metabolite Trimethylamine‐N‐Oxide and Multiple Health Outcomes: An Umbrella Review and Updated Meta‐Analysis.” The American Journal of Clinical Nutrition 116: 230–43. 10.1093/ajcn/nqac074 35348578 PMC9257469

[50] Romano Kymberleigh, A. , I. Vivas Eugenio , Daniel Amador‐Noguez , and E. Rey Federico . 2015. “Intestinal Microbiota Composition Modulates Choline Bioavailability From Diet and Accumulation of the Proatherogenic Metabolite Trimethylamine‐N‐Oxide.” MBio 6: e02481. 10.1128/mbio.02481-14 25784704 PMC4453578

[51] Organ, Chelsea L. , Hiroyuki Otsuka , Shashi Bhushan , Zeneng Wang , Jessica Bradley , Rishi Trivedi , David J. Polhemus , et al. 2016. “Choline Diet and its Gut Microbe‐Derived Metabolite, Trimethylamine N‐Oxide, Exacerbate Pressure Overload‐Induced Heart Failure.” Circulation: Heart Failure 9: e002314. 10.1161/circheartfailure.115.002314 26699388 PMC4943035

[52] Seldin, Marcus M. , Yonghong Meng , Hongxiu Qi , WeiFei Zhu , Zeneng Wang , Stanley L. Hazen , Aldons J. Lusis , and Diana M. Shih . 2016. “Trimethylamine N‐Oxide Promotes Vascular Inflammation Through Signaling of Mitogen‐Activated Protein Kinase and Nuclear Factor‐κB.” Journal of the American Heart Association 5: e002767. 10.1161/jaha.115.002767 26903003 PMC4802459

[53] Sun, Xiaolei , Xuefei Jiao , Yarong Ma , Yong Liu , Lei Zhang , Yanzheng He , Yunhui Chen , and Yunhui Chen . 2016. “Trimethylamine N‐Oxide Induces Inflammation and Endothelial Dysfunction in Human Umbilical Vein Endothelial Cells Via Activating ROS‐TXNIP‐NLRP3 Inflammasome.” Biochemical and Biophysical Research Communications 481: 63–70. 10.1016/j.bbrc.2016.11.017 27833015

[54] Chen, Ming‐liang , Xiao‐hui Zhu , Li Ran , He‐dong Lang , Long Yi , and Man‐tian Mi . 2017. “Trimethylamine‐N‐Oxide Induces Vascular Inflammation by Activating the NLRP3 Inflammasome Through the SIRT3‐SOD2‐mtROS Signaling Pathway.” Journal of the American Heart Association 6: e006347. 10.1161/jaha.117.006347 28871042 PMC5634285

[55] Makrecka‐Kuka, Marina , Kristine Volska , Unigunde Antone , Reinis Vilskersts , Solveiga Grinberga , Dace Bandere , Edgars Liepinsh , and Maija Dambrova . 2017. “Trimethylamine N‐Oxide Impairs Pyruvate and Fatty Acid Oxidation In Cardiac Mitochondria.” Toxicology Letters 267: 32–8. 10.1016/j.toxlet.2016.12.017 28049038

[56] Li, Zehua , Zhiye Wu , Jianyun Yan , Hailin Liu , Qicai Liu , Yi Deng , Caiwen Ou , and Minsheng Chen . 2019. “Gut Microbe‐Derived Metabolite Trimethylamine N‐Oxide Induces Cardiac Hypertrophy and Fibrosis.” Laboratory Investigation 99: 346–57. 10.1038/s41374-018-0091-y 30068915

[57] Brunt, Vienna E. , Rachel A. Gioscia‐Ryan , Abigail G. Casso , Nicholas S. VanDongen , Brian P. Ziemba , Zachary J. Sapinsley , James J. Richey , et al. 2020. “Trimethylamine‐N‐Oxide Promotes Age‐Related Vascular Oxidative Stress and Endothelial Dysfunction in Mice and Healthy Humans.” Hypertension 76: 101–12. 10.1161/hypertensionaha.120.14759 32520619 PMC7295014

[58] Yoshida, Yohko , Ippei Shimizu , Atsuhiro Shimada , Keita Nakahara , Sachiko Yanagisawa , Minoru Kubo , Shinji Fukuda , et al. 2022. “Brown Adipose Tissue Dysfunction Promotes Heart Failure Via a Trimethylamine N‐Oxide‐Dependent Mechanism.” Scientific Reports 12: 14883. 10.1038/s41598-022-19245-x 36050466 PMC9436957

[59] Zhang, L. E. I. , Feifei Xie , Haie Tang , Xinrong Zhang , Jianxia Hu , Xiaohong Zhong , Nirong Gong , et al. 2022. “Gut Microbial Metabolite TMAO Increases Peritoneal Inflammation and Peritonitis Risk in Peritoneal Dialysis Patients.” Translational Research 240: 50–63. 10.1016/j.trsl.2021.10.001 34673277

[60] den Besten, Gijs , Karen van Eunen , Albert K. Groen , Koen Venema , Dirk‐Jan Reijngoud , and Barbara M. Bakker . 2013. “The Role of Short‐Chain Fatty Acids in the Interplay Between Diet, Gut Microbiota, and Host Energy Metabolism.” Journal of Lipid Research 54: 2325–40. 10.1194/jlr.R036012 23821742 PMC3735932

[61] Cook, Sellin . 1998. “Review Article: Short Chain Fatty Acids in Health and Disease.” Alimentary Pharmacology & Therapeutics 12: 499–507. 10.1046/j.1365-2036.1998.00337.x 9678808

[62] Li, Lin , Sen‐jie Zhong , Si‐yuan Hu , Bin Cheng , Hong Qiu , and Zhi‐xi Hu . 2021. “Changes of Gut Microbiome Composition and Metabolites Associated with Hypertensive Heart Failure Rats.” BMC Microbiology 21: 141. 10.1186/s12866-021-02202-5 33952214 PMC8097775

[63] Kirschner, Sarah K. , Nicolaas E. P. Deutz , Iris Rijnaarts , Tiffany J. Smit , Daniel J. Larsen , and Mariëlle P. K. J. Engelen . 2022. “Impaired Intestinal Function is Associated With Lower Muscle and Cognitive Health and Well‐Being in Patients with Congestive Heart Failure.” Journal of Parenteral and Enteral Nutrition 46: 660–70. 10.1002/jpen.2193 34021600

[64] Martin‐Gallausiaux, Camille , Ludovica Marinelli , Hervé M. Blottière , Pierre Larraufie , and Nicolas Lapaque . 2021. “SCFA: Mechanisms and Functional Importance in the Gut.” Proceedings of the Nutrition Society 80: 37–49. 10.1017/S0029665120006916 32238208

[65] Donohoe, Dallas R. , Leonard B. Collins , Aminah Wali , Rebecca Bigler , Wei Sun , and Scott J. Bultman . 2012. “The Warburg Effect Dictates the Mechanism of Butyrate‐Mediated Histone Acetylation and Cell Proliferation.” Molecular Cell 48: 612–26. 10.1016/j.molcel.2012.08.033 23063526 PMC3513569

[66] Mathewson, Nathan D. , Robert Jenq , Anna V. Mathew , Mark Koenigsknecht , Alan Hanash , Tomomi Toubai , Katherine Oravecz‐Wilson , et al. 2016. “Gut Microbiome‐Derived Metabolites Modulate Intestinal Epithelial Cell Damage and Mitigate Graft‐Versus‐Host Disease.” Nature Immunology 17: 505–13. 10.1038/ni.3400 26998764 PMC4836986

[67] Bartolomaeus, Hendrik , András Balogh , Mina Yakoub , Susanne Homann , Lajos Sascha Höges , Dmitry Tsvetkov , Alexander Krannich , et al. 2019. “Short‐Chain Fatty Acid Propionate Protects From Hypertensive Cardiovascular Damage.” Circulation 139: 1407–21. 10.1161/circulationaha.118.036652 30586752 PMC6416008

[68] Kaye, David M. , Waled A. Shihata , Hamdi A. Jama , Kirill Tsyganov , Mark Ziemann , Helen Kiriazis , Duncan Horlock , et al. 2020. “Deficiency of Prebiotic Fiber and Insufficient Signaling Through Gut Metabolite‐Sensing Receptors Leads to Cardiovascular Disease.” Circulation 141: 1393–403. 10.1161/circulationaha.119.043081 32093510

[69] Li, Meng , Betty C. A. M. van Esch , Gerry T. M. Wagenaar , Johan Garssen , Gert Folkerts , and Paul A. J. Henricks . 2018. “Pro‐ and Anti‐Inflammatory Effects of Short Chain Fatty Acids On Immune and Endothelial Cells.” European Journal of Pharmacology 831: 52–9. 10.1016/j.ejphar.2018.05.003 29750914

[70] Chen, An‐tian , Chen‐yu Wang , Wen‐ling Zhu , and Wei Chen . 2022. “Coagulation Disorders and Thrombosis in COVID‐19 Patients and a Possible Mechanism Involving Endothelial Cells: A Review.” Aging and Disease 13: 144–56. 10.14336/ad.2021.0704 35111367 PMC8782553

[71] He, Jin , Peiwen Zhang , Linyuan Shen , Lili Niu , Ya Tan , Lei Chen , Ye Zhao , et al. 2020. “Short‐Chain Fatty Acids and their Association with Signalling Pathways in Inflammation, Glucose and Lipid Metabolism.” International Journal of Molecular Sciences 21: 6356. 10.3390/ijms21176356 32887215 PMC7503625

[72] Masui, Ryuta , Makoto Sasaki , Yasushi Funaki , Naotaka Ogasawara , Mari Mizuno , Akihito Iida , Shinya Izawa , et al. 2013. “G Protein‐Coupled Receptor 43 Moderates Gut Inflammation Through Cytokine Regulation From Mononuclear Cells.” Inflammatory Bowel Diseases 19: 2848–56. 10.1097/01.MIB.0000435444.14860.ea 24141712

[73] Vinolo, Marco A. R. , Hosana G. Rodrigues , Elaine Hatanaka , Fábio T. Sato , Sandra C. Sampaio , and Rui Curi . 2011. “Suppressive Effect of Short‐Chain Fatty Acids on Production of Proinflammatory Mediators by Neutrophils.” The Journal of Nutritional Biochemistry 22: 849–55. 10.1016/j.jnutbio.2010.07.009 21167700

[74] Ohira, Hideo , Yoshio Fujioka , Chikae Katagiri , Rie Mamoto , Michiko Aoyama‐Ishikawa , Katsumi Amako , Yoshihiro Izumi , et al. 2013. “Butyrate Attenuates Inflammation and Lipolysis Generated by the Interaction of Adipocytes and Macrophages.” Journal of Atherosclerosis and Thrombosis 20: 425–42. 10.5551/jat.15065 23470566

[75] Halnes, Isabel , Katherine J. Baines , Bronwyn S. Berthon , Lesley K. MacDonald‐Wicks , Peter G. Gibson , and Lisa G. Wood . 2017 “Soluble Fibre Meal Challenge Reduces Airway Inflammation and Expression of GPR43 and GPR41 in Asthma.” Nutrients 9: 57. 10.3390/nu9010057 28075383 PMC5295101

[76] Vinolo, Marco A. R. , Hosana G. Rodrigues , Renato T. Nachbar , and Rui Curi . 2011. “Regulation of Inflammation by Short Chain Fatty Acids.” Nutrients 3: 858–76. 10.3390/nu3100858 22254083 PMC3257741

[77] Li, Meng , Betty C. A. M. van Esch , Paul A. J. Henricks , Gert Folkerts , and Johan Garssen . 2018. “The Anti‐Inflammatory Effects of Short Chain Fatty Acids on Lipopolysaccharide‐ or Tumor Necrosis Factor α‐Stimulated Endothelial Cells Via Activation of GPR41/43 and Inhibition of HDACs.” Frontiers in Pharmacology 9: 533. 10.3389/fphar.2018.00533 29875665 PMC5974203

[78] Li, Bo , Xinglishang He , Hai‐Ying Jin , Hui‐Ying Wang , Fu‐Chen Zhou , Ning‐Yu Zhang , Dong‐Ying Jie , et al. 2021. “Beneficial Effects of *Dendrobium Officinale* on Metabolic Hypertensive Rats by Triggering the Enteric‐Origin SCFA‐GPCR43/41 Pathway.” Food & Function 12: 5524–38. 10.1039/d0fo02890h 34002173

[79] Amabebe, Emmanuel , Faith O. Robert , Tarimoboere Agbalalah , and Ebiowei S. F. Orubu . 2020. “Microbial Dysbiosis‐Induced Obesity: Role of Gut Microbiota in Homoeostasis of Energy Metabolism.” British Journal of Nutrition 123: 1127–37. 10.1017/s0007114520000380 32008579

[80] Rees, Douglas . 2017. “The Obesity Epidemic and Our Gut Microbiome–Could it All be Down to Our ‘Bugs’?” The Biochemist 39: 26–9. 10.1042/bio03902026

[81] Chakraborti, Chandra Kanti . 2015. “New‐Found Link Between Microbiota and Obesity.” World Journal of Gastrointestinal Pathophysiology 6: 110–19. 10.4291/wjgp.v6.i4.110 26600968 PMC4644874

[82] Carvalho, Bruno Melo , and Mario Jose Abdalla Saad . 2013. “Influence of Gut Microbiota on Subclinical Inflammation and Insulin Resistance.” Mediators of Inflammation 2013: 986734. 10.1155/2013/986734 23840101 PMC3694527

[83] Davis, Cindy D. 2016. “The Gut Microbiome and its Role in Obesity.” Nutrition Today 51: 167–74. 10.1097/nt.0000000000000167 27795585 PMC5082693

[84] Nemet, Ina , Prasenjit Prasad Saha , Nilaksh Gupta , Weifei Zhu , Kymberleigh A. Romano , Sarah M. Skye , Tomas Cajka , et al. 2020. “A Cardiovascular Disease‐Linked Gut Microbial Metabolite Acts Via Adrenergic Receptors.” Cell 180: 862–877.e822. 10.1016/j.cell.2020.02.016. 32142679 PMC7402401

[85] Zong, Xiao , Qin Fan , Qian Yang , Roubai Pan , Lingfang Zhuang , and Rong Tao . 2022. “Phenylacetylglutamine as a Risk Factor and Prognostic Indicator of Heart Failure.” ESC Heart Failure 9: 2645–53. 10.1002/ehf2.13989 35624536 PMC9288759

[86] Fu, Hui , Bin Kong , Jun Zhu , He Huang , and Wei Shuai . 2023. “Phenylacetylglutamine Increases the Susceptibility of Ventricular Arrhythmias in Heart Failure Mice by Exacerbated Activation of the TLR4/AKT/mTOR Signaling Pathway.” International Immunopharmacology 116: 109795. 10.1016/j.intimp.2023.109795 36736224

[87] David, Lawrence A. , Corinne F. Maurice , Rachel N. Carmody , David B. Gootenberg , Julie E. Button , Benjamin E. Wolfe , Alisha V. Ling , et al. 2014. “Diet Rapidly and Reproducibly Alters the Human Gut Microbiome.” Nature 505: 559–63. 10.1038/nature12820 24336217 PMC3957428

[88] Filippou, Christina D. , Costas P. Tsioufis , Costas G. Thomopoulos , Costas C. Mihas , Kyriakos S. Dimitriadis , Lida I. Sotiropoulou , Christina A. Chrysochoou , Petros I. Nihoyannopoulos , and Dimitrios M. Tousoulis . 2020. “Dietary Approaches to Stop Hypertension (DASH) Diet and Blood Pressure Reduction in Adults With and Without Hypertension: A Systematic Review and Meta‐Analysis of Randomized Controlled Trials.” Advances in Nutrition 11: 1150–60. 10.1093/advances/nmaa041 32330233 PMC7490167

[89] Sacks, Frank M. , Laura P. Svetkey , William M. Vollmer , Lawrence J. Appel , George A. Bray , David Harsha , Eva Obarzanek , et al. 2001. “Effects on Blood Pressure of Reduced Dietary Sodium and the Dietary Approaches to Stop Hypertension (DASH) Diet.” New England Journal of Medicine 344: 3–10. 10.1056/nejm200101043440101 11136953

[90] Ibsen, Daniel B. , Emily B. Levitan , Agneta Åkesson , Bruna Gigante , and Alicja Wolk . 2022. “The DASH Diet is Associated with a Lower Risk of Heart Failure: A Cohort Study.” European Journal of Preventive Cardiology 29: 1114–23. 10.1093/eurjpc/zwac003 34983068

[91] Rifai, Luay , Carol Pisano , Janel Hayden , Suela Sulo , and Marc A. Silver . 2015. “Impact of the DASH Diet on Endothelial Function, Exercise Capacity, and Quality of Life in Patients with Heart Failure.” Baylor University Medical Center Proceedings 28: 151–56. 10.1080/08998280.2015.11929216 25829641 PMC4365107

[92] Kris‐Etherton, Penny , Robert H. Eckel , Barbara V. Howard , Sachiko St. Jeor , and Terry L. Bazzarre . 2001. “Lyon Diet Heart Study: Benefits of a Mediterranean‐Style, National Cholesterol Education Program/American Heart Association Step I Dietary Pattern on Cardiovascular Disease.” Circulation 103: 1823–25. 10.1161/01.cir.103.13.1823 11282918

[93] Lopez‐Garcia, Esther , Fernando Rodriguez‐Artalejo , Tricia Y. Li , Teresa T. Fung , Shanshan Li , Walter C. Willett , Eric B. Rimm , and Frank B. Hu . 2014. “The Mediterranean‐Style Dietary Pattern and Mortality Among Men and Women with Cardiovascular Disease.” The American Journal of Clinical Nutrition 99: 172–80. 10.3945/ajcn.113.068106 24172306 PMC3862454

[94] Papadaki, Angeliki , Miguel Ángel Martínez‐González , Angel Alonso‐Gómez , Javier Rekondo , Jordi Salas‐Salvadó , Dolores Corella , Emilio Ros , et al. 2017. “Mediterranean Diet and Risk of Heart Failure: Results from the PREDIMED Randomized Controlled Trial.” European Journal of Heart Failure 19: 1179–85. 10.1002/ejhf.750 28133855

[95] Sanches Machado d'Almeida, Karina , Stefanny Ronchi Spillere , Priccila Zuchinali , and Gabriela Corrêa Souza . 2018. “Mediterranean Diet and Other Dietary Patterns in Primary Prevention of Heart Failure and Changes in Cardiac Function Markers: A Systematic Review.” Nutrients 10: 58. 10.3390/nu10010058 29320401 PMC5793286

[96] Hill, Colin , Francisco Guarner , Gregor Reid , Glenn R. Gibson , Daniel J. Merenstein , Bruno Pot , Lorenzo Morelli , et al. 2014. “Expert Consensus Document. The International Scientific Association for Probiotics and Prebiotics Consensus Statement on the Scope and Appropriate Use of the Term Probiotic.” Nature Reviews Gastroenterology & Hepatology 11: 506–14. 10.1038/nrgastro.2014.66 24912386

[97] Gan, Xiaohong Tracey , Grace Ettinger , Cathy X. Huang , Jeremy P. Burton , James V. Haist , Venkatesh Rajapurohitam , James E. Sidaway , et al 2014. “Probiotic Administration Attenuates Myocardial Hypertrophy and Heart Failure After Myocardial Infarction in the Rat.” Circulation: Heart Failure 7: 491–9. 10.1161/circheartfailure.113.000978 24625365

[98] Karim, Asima , Tahir Muhammad , Islam Shah , Javaidullah Khan , and Rizwan Qaisar . 2022. “A Multistrain Probiotic Reduces Sarcopenia By Modulating Wnt Signaling Biomarkers in Patients With Chronic Heart Failure.” Journal of Cardiology 80: 449–55. 10.1016/j.jjcc.2022.06.006 35750555

[99] Costanza, Annelise C. , Samuel D. Moscavitch , Hugo C. C. Faria Neto , and Evandro T. Mesquita . 2015. “Probiotic Therapy With *Saccharomyces Boulardii* for Heart Failure Patients: A Randomized, Double‐Blind, Placebo‐Controlled Pilot Trial.” International Journal of Cardiology 179: 348–50. 10.1016/j.ijcard.2014.11.034 25464484

[100] Pourrajab, Behnaz , Nasim Naderi , Leila Janani , Marjan Hajahmadi , Vahid Mofid , Afsaneh Dehnad , Mohammad Hassan Sohouli , Sharieh Hosseini , and Farzad Shidfar . 2022. “The Impact of Probiotic Yogurt Versus Ordinary Yogurt on Serum sTWEAK, sCD163, ADMA, LCAT and BUN in Patients with Chronic Heart Failure: A Randomized, Triple‐Blind, Controlled Trial.” Journal of the Science of Food and Agriculture 102: 6024–35. 10.1002/jsfa.11955 35460085

[101] Awoyemi, Ayodeji , Cristiane Mayerhofer , Alex S. Felix , Johannes R. Hov , Samuel D. Moscavitch , Knut Tore Lappegård , Anders Hovland , et al. 2021. “Rifaximin or *Saccharomyces Boulardii* in Heart Failure with Reduced Ejection Fraction: Results From the Randomized GutHeart Trial.” EBioMedicine 70: 103511. 10.1016/j.ebiom.2021.103511 34329947 PMC8339250

[102] Gupta, Arjun , and Sahil Khanna . 2017. “Fecal Microbiota Transplantation.” JAMA 318: 102. 10.1001/jama.2017.6466 28672320

[103] Jia, Qiujin , Hao Li , Huan Zhou , Xiaonan Zhang , Ao Zhang , Yingyu Xie , Yanyang Li , Shichao Lv , and Junping Zhang . 2019. “Role and Effective Therapeutic Target of Gut Microbiota in Heart Failure.” Cardiovascular Therapeutics 2019: 1–10. 10.1155/2019/5164298 PMC6885196 31819762

[104] Wilson, Brooke C. , Tommi Vatanen , Wayne S. Cutfield , and Justin M. O'Sullivan . 2019. “The Super‐Donor Phenomenon in Fecal Microbiota Transplantation.” Frontiers in Cellular and Infection Microbiology 9: 2. 10.3389/fcimb.2019.00002 30719428 PMC6348388

[105] Chehoud, Christel , Anatoly Dryga , Young Hwang , Dorottya Nagy‐Szakal , Emily B. Hollister , Ruth Ann Luna , James Versalovic , Richard Kellermayer , and Frederic D. Bushman . 2016. “Transfer of Viral Communities Between Human Individuals During Fecal Microbiota Transplantation.” MBio 7: e00322. 10.1128/mBio.00322-16 27025251 PMC4817255

[106] Zhou, Xin , Jing Li , Junli Guo , Bin Geng , Wenjie Ji , Qian Zhao , Jinlong Li , et al. 2018. “Gut‐Dependent Microbial Translocation Induces Inflammation and Cardiovascular Events After ST‐Elevation Myocardial Infarction.” Microbiome 6: 66. 10.1186/s40168-018-0441-4 29615110 PMC5883284

[107] Ponziani, Francesca Romana , Maria Assunta Zocco , Francesca D'Aversa , Maurizio Pompili , and Antonio Gasbarrini . 2017“Eubiotic Properties of Rifaximin: Disruption of the Traditional Concepts in Gut Microbiota Modulation.” World Journal of Gastroenterology 23: 4491–9. 10.3748/wjg.v23.i25.4491 28740337 PMC5504364

[108] Chen, Ming‐liang , Long Yi , Yong Zhang , Xi Zhou , Li Ran , Jining Yang , Jun‐dong Zhu , Qian‐yong Zhang , and Man‐tian Mi . 2016. “Resveratrol Attenuates Trimethylamine‐N‐Oxide (TMAO)‐Induced Atherosclerosis by Regulating TMAO Synthesis and Bile Acid Metabolism Via Remodeling of the Gut Microbiota.” MBio 7: e02210–5. 10.1128/mBio.02210-15 27048804 PMC4817264

[109] Barrea, Luigi , Giovanna Muscogiuri , Giuseppe Annunziata , Daniela Laudisio , Giulia de Alteriis , Gian Carlo Tenore , Annamaria Colao , and Silvia Savastano . 2019. “A New Light on Vitamin D in Obesity: A Novel Association With Trimethylamine‐N‐Oxide (TMAO).” Nutrients 11: 1310. 10.3390/nu11061310 31185686 PMC6627576

[110] Obeid, Rima , Hussain M. Awwad , Susanne H. Kirsch , Christiane Waldura , Wolfgang Herrmann , Stefan Graeber , and Juergen Geisel . 2017. “Plasma Trimethylamine‐N‐Oxide Following Supplementation with Vitamin D or D Plus B Vitamins.” Molecular Nutrition & Food Research 61: 1600358. 10.1002/mnfr.201600358 27569255

[111] Wang, Guangji , Bin Kong , Wei Shuai , Hui Fu , Xiaobo Jiang , and He Huang . 2020. “3,3‐Dimethyl‐1‐butanol Attenuates Cardiac Remodeling in Pressure‐Overload‐Induced Heart Failure Mice.” The Journal of Nutritional Biochemistry 78: 108341. 10.1016/j.jnutbio.2020.108341 32004931

[112] Feng, Wuwen , Hui Ao , Cheng Peng , and Dan Yan . 2019. “Gut Microbiota, A New Frontier to Understand Traditional Chinese Medicines.” Pharmacological Research 142: 176–91. 10.1016/j.phrs.2019.02.024 30818043

